# Vicarious praise and pain: parental neural responses to social feedback about their adolescent child

**DOI:** 10.1093/scan/nsab004

**Published:** 2021-01-12

**Authors:** Lisanne A E M van Houtum, Mirjam C M Wever, Loes H C Janssen, Charlotte C van Schie, Geert-Jan Will, Marieke S Tollenaar, Bernet M Elzinga

**Affiliations:** Department of Clinical Psychology, Institute of Psychology, Leiden University, Leiden, South Holland 2300 RB, The Netherlands; Leiden Institute for Brain and Cognition, Leiden University, Leiden, South Holland 2300 RC, The Netherlands; Department of Clinical Psychology, Institute of Psychology, Leiden University, Leiden, South Holland 2300 RB, The Netherlands; Leiden Institute for Brain and Cognition, Leiden University, Leiden, South Holland 2300 RC, The Netherlands; Department of Clinical Psychology, Institute of Psychology, Leiden University, Leiden, South Holland 2300 RB, The Netherlands; Leiden Institute for Brain and Cognition, Leiden University, Leiden, South Holland 2300 RC, The Netherlands; Department of Clinical Psychology, Institute of Psychology, Leiden University, Leiden, South Holland 2300 RB, The Netherlands; Leiden Institute for Brain and Cognition, Leiden University, Leiden, South Holland 2300 RC, The Netherlands; Illawarra Health and Medical Research Institute and School of Psychology, University of Wollongong, Wollongong, NSW 2522, Australia; Department of Clinical Psychology, Institute of Psychology, Leiden University, Leiden, South Holland 2300 RB, The Netherlands; Leiden Institute for Brain and Cognition, Leiden University, Leiden, South Holland 2300 RC, The Netherlands; Department of Clinical Psychology, Institute of Psychology, Leiden University, Leiden, South Holland 2300 RB, The Netherlands; Leiden Institute for Brain and Cognition, Leiden University, Leiden, South Holland 2300 RC, The Netherlands; Department of Clinical Psychology, Institute of Psychology, Leiden University, Leiden, South Holland 2300 RB, The Netherlands; Leiden Institute for Brain and Cognition, Leiden University, Leiden, South Holland 2300 RC, The Netherlands

**Keywords:** social feedback, vicarious praise and criticism, functional magnetic resonance imaging (fMRI), parental perceptions, parent–child relationship

## Abstract

Social feedback, such as praise or critique, profoundly impacts our mood and social interactions. It is unknown, however, how *parents* experience praise and critique about their child and whether their mood and neural responses to such ‘vicarious’ social feedback are modulated by parents’ perceptions of their child. Parents (*n* = 60) received positive, intermediate and negative feedback words (i.e. personality characteristics) about their adolescent child during a magnetic resonance imaging scan. After each word, parents indicated their mood. After positive feedback their mood improved and activity in ventromedial prefrontal cortex and posterior cingulate cortex/precuneus increased. Negative feedback worsened parents’ mood, especially when perceived as inapplicable to their child, and increased activity in anterior cingulate cortex, anterior insula, dorsomedial prefrontal cortex and precuneus. Parents who *generally* viewed their child more positively showed amplified mood responses to both positive and negative feedback and increased activity in dorsal striatum, inferior frontal gyrus and insula in response to negative feedback. These findings suggest that vicarious feedback has similar effects and engages similar brain regions as observed during feedback about the self and illustrates this is dependent on parents’ beliefs of their child’s qualities and flaws. Potential implications for parent–child dynamics and children’s own self-views are discussed.

## Introduction

Social feedback, such as praise or criticism, provides valuable insights into the way one is viewed by others (van Schie *et al.*, 2018). For parents, it is a common experience to receive social feedback about their child, for example during conversations with teachers, sport coaches, clinicians, friends or family members (e.g. Pillet-Shore, 2012, 2015). It is to be expected that parents tend to empathize with their child’s feelings when their child is being socially judged or evaluated, given their genetic ties and their large effort of investment (Brummelman *et al.*, 2015). Parents may also feel personally judged, as the feedback potentially touches their own identity, values, parenting skills and/or competencies (Thai *et al.*, 2019). As a result, parents might vicariously experience and be genuinely affected by social feedback about their child. However, little is known about the affective and underlying neural signatures of these experiences. Excessive responses to feedback about the child may vitally shape interpersonal dynamics of parent *and* child (e.g. in the context of teacher or sport coach evaluations). Eventually, parental reactions to feedback may thus also impact on how children view themselves (Lee *et al.*, 2017). Therefore, this study examined how parents react—both on an affective and a neural level—to praise and critique about their child, i.e. ‘vicarious’ social feedback (social feedback received about others).

While brain regions supporting vicarious feedback processing have received relatively little attention, brain regions involved in the processing of social feedback about the *self* have been extensively studied. Receiving positive feedback has been consistently associated with increased activation in the ventral striatum (VS) and ventromedial prefrontal cortex (vmPFC; Izuma *et al.*, 2008; Davey *et al.*, 2010; Gunther Moor *et al.*, 2010; Korn *et al.*, 2012; Morelli *et al.*, 2015; Muscatell *et al.*, 2016; Will *et al.*, 2017; Kawamichi *et al.*, 2018; Schindler *et al.*, 2019; Will *et al*., 2020). Receiving negative social feedback, in contrast, has been associated with increased activation in anterior cingulate cortex (ACC) and anterior insula (AI; Eisenberger *et al.*, 2011; Cacioppo *et al.*, 2013; Rotge *et al.*, 2015; Muscatell *et al.*, 2016; Will *et al.*, 2016; Kawamichi *et al.*, 2018; van Schie *et al.*, 2018; Schindler *et al.*, 2019; Fritz *et al.*, 2020). There is emerging evidence that sub-regions of ACC and AI also respond to positive social feedback, suggesting that these regions may process saliency of social evaluation rather than negative affect associated with negative feedback *per se* (Achterberg *et al.*, 2016; Dalgleish *et al.*, 2017; van Schie *et al.*, 2018). Finally, being socially evaluated by others elicits activity in brain regions important for mentalizing (i.e. understanding the mental states of others) including dorsomedial PFC (dmPFC), precuneus and temporoparietal junction (TPJ; Van Overwalle, 2009; Schurz *et al.*, 2014; Molenberghs *et al.*, 2016; Muscatell *et al.*, 2016; Kawamichi *et al.*, 2018; van Schie *et al.*, 2018). Based on previous research showing that self- and close-other-processing can engage (or suppress) similar neural circuitries (Murray *et al.*, 2012; Heleven and Van Overwalle, 2019), we hypothesize that the brain regions responding to social feedback directed at the self may also be involved in parental *vicarious* social feedback processing.

It is likely that affective and neural responses to vicarious feedback are modulated by parental pre-existing beliefs about their child’s qualities and flaws. Therefore, it is crucial to not only examine how feedback valence (i.e. positive and negative feedback) modulates neural responses to feedback, but also the *consistency* of feedback with existing parental perceptions of their child. In general, social feedback that is consistent with one’s own views is processed more easily and experienced as more pleasant, as this feedback confirms one’s perceptions as opposed to feedback that is incompatible with self-views (Stinson *et al.*, 2010; van Schie *et al.*, 2018). For example, van Schie *et al.* (2018) showed that receiving feedback words that were rated as more applicable to the self elicited more positive mood and increased activation in left precuneus, both for positive and negative feedback. Particularly negative feedback words that were considered *in*applicable (i.e. perceived ‘misplaced criticism’) had a detrimental impact on mood (van Schie *et al.*, 2018). It remains to be investigated how such findings translate to vicarious feedback about one’s child, that is, how feedback that is inconsistent with parental perceptions of their child might be processed differently than feedback that is consistent with parents’ perceptions.

Generally, parents view their children through rosy glasses and overestimate their qualities (Taylor and Brown, 1988; Wenger and Fowers, 2008; Brummelman *et al.*, 2015). For example, parents rate positive trait characteristics as relatively more and negative characteristics as less descriptive of their child, compared to another child of the same age (Cohen and Fowers, 2004; Wenger and Fowers, 2008). However, parents may differ from one another with respect to this ‘better-than-average effect’ (Alicke *et al.*, 1995), i.e. some parents tend to hold positive views of their child that are grounded in reality, whereas other parents hold *overly* positive views and some even have overly negative views (Brummelman *et al.*, 2015). When parents have a general tendency to view their child in a more positive light, they may show more intense affective reactions to vicarious feedback about their child, both in response to criticism and praise, as they are highly motivated to maintain their favourable view (Alicke *et al.*, 2020). In contrast, parents with a neutral or relatively negative view may be less affected by both negative and positive feedback.

The goals of this study are hence threefold: First, we examine affective and neural responses of parents to praise and critique about their child (i.e. responses to positive, intermediate and negative feedback words). Second, we investigate how (in)consistency of feedback words with parents’ own perceptions of their child (i.e. (in)applicability) impacts parental affective and neural responses to vicarious social feedback, and third, how parents’ *general* view of their child impacts these responses.

We hypothesize that positive feedback about their child, compared to intermediate and negative feedback, will be associated with increases in parental mood, whereas negative feedback will be associated with decreases in mood. Based on work examining neural responses to social feedback directed at the self, we expect increased activity in VS and vmPFC in response to positive feedback (Izuma *et al.*, 2008; Davey *et al.*, 2010; Gunther Moor *et al.*, 2010; Korn *et al.*, 2012; Morelli *et al.*, 2015; Muscatell *et al.*, 2016; Will *et al.*, 2017, Kawamichi *et al.*, 2018; Schindler *et al.*, 2019; Will *et al.*, 2020) and increased activity in ACC and AI in response to negative feedback about the child (Eisenberger *et al.*, 2011; Cacioppo *et al.*, 2013; Rotge *et al.*, 2015; Muscatell *et al.*, 2016; Will *et al.*, 2016; Kawamichi *et al.*, 2018; van Schie *et al.*, 2018; Schindler *et al.*, 2019; Fritz *et al.*, 2020). We expect that brain regions important for mentalizing (e.g. dmPFC, precuneus and TPJ) will be activated both in response to positive and negative feedback compared to intermediate feedback (Muscatell *et al.*, 2016; Kawamichi *et al.*, 2018; van Schie *et al.*, 2018). Based on van Schie *et al.* (2018), we expect that social feedback words consistent with parental perceptions of their child (i.e. more applicable feedback) will result in improved mood regardless of feedback valence and that especially inapplicable negative feedback words (perceived ‘misplaced criticism’) will negatively impact parents’ mood. Lastly, we expect that *generally* viewing the child more positively is related to more intense affective responses to both positive and negative feedback.

## Methods

### Participants

Parents and adolescents participated in a Dutch multi-method two-generation study called RE-PAIR (‘Relations and Emotions in Parent-Adolescent Interaction Research’), investigating the bidirectional interplay between parent–adolescent interactions and adolescent mental well-being. Analyses in the current paper are restricted to parents of non-clinical adolescents. Inclusion criteria for non-clinical adolescents in the RE-PAIR study were age between 11 and 17 years at the time of the first assessment day (i.e. lab session), having started secondary school, living with one or both parents and no diagnosis of major depressive disorder or dysthymia in their lifetime or any other mental health problem in the 2 years preceding study participation [assessed using Kiddie-Schedule for Affective Disorders and Schizophrenia-Present and Lifetime Version (K-SADS-PL; Kaufman *et al.*, 1996)]. For parents, no inclusion or exclusion criteria were specified, except for a good command of Dutch language. For the functional magnetic resonance imaging (fMRI) part of the study (i.e. scan session), only one parent per family could participate and MRI incompatibility (i.e. implanted medical devices, non-removable metal in the body, pregnancy and claustrophobia) was specified as exclusion criterion for both parents and adolescents.

In total, 63 parents took part in the scan session. Three parents were excluded due to sleep apnoea (*n *= 1), brain abnormalities (*n *= 1) and misinterpretation of task instructions (*n *= 1), resulting in a final sample of 60 parents; see Table 1 for descriptive statistics (for more sample details, see Supplementary Material 1).

**Table 1.** Participants’ demographics and descriptive statistics (*n *= 60)

Variables	All parents (*n* = 60)	Mothers (*n* = 35)	Fathers (*n* = 25)	Gender differences		
	Mean (s.d.)/*n* (%)	Mean (s.d.)/*n* (%)	Mean (s.d.)/*n* (%)	*t/U*	*df*	*P*
Age (years)	49.2 (4.71)	47.6 (4.31)	51.5 (4.33)	−3.52	58	<0.001***
Gender child, *n* male (%)	22 (36.7)	11 (31.4)	11 (44.0)			
Age child (years)	16.2 (1.21)	16.5 (0.97)	15.7 (1.40)	565^1^		0.057, ns
Educational level, *n* (%)						
Vocational (MBO)	19 (31.7)	12 (34.3)	7 (28.0)			
Higher (HBO/University)	41 (68.3)	23 (65.7)	18 (72.0)			
Handedness (EHI score)	76.2 (55.2)	72.1 (57.6)	81.8 (52.4)	2.00	58	0.506, ns
Right-handed, *n* (%)	54 (90.0)	31 (88.6)	23 (92.0)			
General view of child	1.03 (0.55)	0.99 (0.56)	1.09 (0.55)	−0.69	58	0.493, ns

*Notes*: ^1^As equal variances were not assumed, a nonparametric Mann–Whitney U-test was conducted. *** *P *< 0.001. EHI, Edinburgh Handedness Inventory; HBO, higher education (in Dutch: Hoger beroepsonderwijs); MBO, vocational training (in Dutch: Middelbaar beroepsonderwijs).

The study was approved by the Medical Ethics Review Committee of Leiden University Medical Centre (LUMC; reference: P17.241; protocol: NL62502.058.17). The study was conducted in accordance with the Dutch Medical Research Involving Human Subjects Act (WMO) and Declaration of Helsinki.

### Procedure

After initial phone screening, families filled out several questionnaires and were invited for the lab session. During this session, written informed consent was obtained, after which families performed several tasks, including video-recorded interaction tasks [i.e. in order: a problem-solving task (Sheeber *et al.*, 2007), an event-planning task (Schwartz *et al.*, 2012) and a reminiscence task (Sheeber *et al.*, 2012) to elicit positive as well as negative emotions and a wide range of parent–child interactions] and questionnaires, including questions about personality characteristics of their child. After the lab session, families completed an ecological momentary assessment diary for 14 consecutive days (to be reported elsewhere; Janssen *et al.*, in prep.). Moreover, adolescents and one of their parents were invited for the scan session at the LUMC, which was scheduled at least 1 week after the lab session (*M* = 7.58 weeks, s.d. = 6.56, range: 1.86–37.86). During this session, participants provided written informed consent again, were accustomed to the scanning environment by means of a mock scanner and received detailed instructions about the tasks. Before the actual scanning procedure, parents filled out several questionnaires, including the Edinburgh Handedness Inventory (EHI; Oldfield, 1971). Parents performed three tasks in an MRI scanner [i.e. in order: an eye-contact task, a parental empathy task (see Wever *et al.*, submitted) and the vicarious social feedback task as described here]. Upon completion of scanning, participants were fully debriefed about the cover story and given the opportunity to ask questions. The task was well-received by parents, and all parents were positive about the study during the debriefing. Parents received a €30 recompense for the scan session and travel expenses were reimbursed.

### Materials

#### Vicarious social feedback task.

The vicarious social feedback task was based on a social feedback task previously developed in our lab to investigate the neural correlates of social feedback directed toward the self (van Schie *et al.*, 2018). In this modified version, parents received positive, intermediate and negative feedback about their child (in the form of words describing personality characteristics) supposedly given by research team members, based on observations of recorded parent–adolescent interaction videos (during a preceding lab session).

During the first lab session, parents performed three different video-recorded interaction tasks with their child during which a large variety of emotional and personally relevant topics and events were extensively discussed. Furthermore, parents had rated 49 feedback words in terms of valence (‘What do you think of this personality characteristic?’) on a scale of −4 (‘very negative’) to 0 (‘neutral’) to 4 (‘very positive’) and in terms of applicability to their child (‘To what extent does this personality characteristic apply to your child?’) on a scale of 1 (‘not at all’) to 5 (‘very much’).

During the scan session (at least 1 week later than the first lab session)—prior to performing the task in the scanner—parents were informed that several research assistants were asked to judge their child in the previously recorded interaction videos and to choose both positive and negative personality characteristics that best describe their child from a provided list of feedback words. We suggested that the feedback was based on observations of their child during these interactions, to ensure that it was credible for parents that the feedback was based on a wide range of observations of their child. In reality, each parent received the same (fake) feedback, split in three predetermined and validated valence categories: 15 positive (e.g. ‘Respectful’), 15 intermediate (e.g. ‘Reserved’) and 15 negative words (e.g. ‘Mean’; see Supplementary Material 2). These feedback words were presented in a semi-randomized fashion, such that consecutive feedback words were not of similar valence. To start and finish on a positive note, the task started and ended with two positive feedback fillers (i.e. not included in the analyses), in a fixed order.

**Fig. 1.**
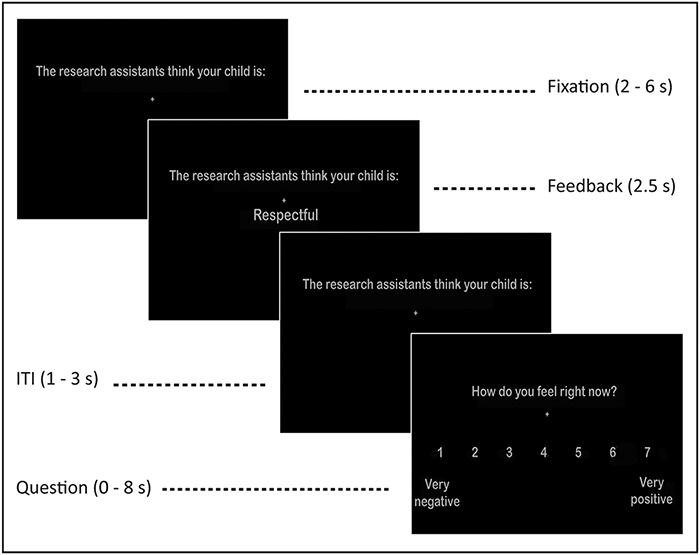
Trial structure of vicarious social feedback task.

Each trial started with a jittered fixation cross with a uniformly distributed duration varying between 2 and 6 s (*M* = 4 s). Then, a feedback word was displayed on the screen for 2.5 s, with a jittered inter-trial-interval fixation cross varying between 1 and 3 s (*M* = 2 s). The sentence ‘The research assistants think that your child is:’ was shown on the screen during each trial. Following each feedback word, parents rated their current mood (‘How do you feel right now?’) on a scale of 1 (‘very negative’) to 7 (‘very positive’) using MR-compatible button boxes. Participants used their left index and middle fingers to move from left to right on the scale and their right index finger to confirm their responses. The mood question was self-paced and lasted for a maximum of 8 s (see Figure 1). If participants failed to respond within the timeframe, the message ‘Too late’ was displayed for 1 s and the trial was excluded from analyses (total excluded trials: *n* = 2).

Following the scans, parents were fully debriefed, by explaining the purpose of the study and the reason for manipulation. This included a manipulation check interview (see Supplementary Material 3). The majority of parents (*n* = 50, 83.3%) were categorized as believing the cover story that research assistants provided the feedback about their child. Additionally, participants received a letter in which we explained the experimental set-up and we asked if they would like to be called 3 days later to evaluate their experiences.

The task was programmed in E-Prime 2.0 (Psychological Software Tools, Pittsburgh, PA) and presented on a BOLD screen, which participants could see via a mirror attached to the head coil.

#### Parents’ general view of their child.

We calculated parents’ general tendency to view their child positively by multiplying parents’ applicability ratings with *z*-scored valence ratings of feedback words. We averaged these applicability × *z*-scored valence values over all feedback words per participant to create a *general* view score. For each parent, a higher score indicated overall more positive (‘rosy’) views of their child (i.e. many positive feedback words were rated as applicable and many negative feedback words as inapplicable). Although the possible range was −7.5 to 7.5, the observed range was −0.35 to 2.12 (see Table 1), following a normal distribution, demonstrating that parents on average have a relatively positive view of their child, but also clear inter-individual differences.

#### MRI data acquisition.

MRI scans were acquired using a Philips Achieva dStream 3.0-Tesla scanner (Philips Medical Systems, Best, NL) equipped with a SENSE-32 whole-head coil. Head motion was restricted using foam inserts. Functional scans were collected with T2*-weighted echo-planar imaging sequence [TR (repetition time): 2.2 s, TE (echo time): 30 ms, flip angle: 80°; 38 transverse slices (anterior-to-posterior); FOV (field-of-view): 220 × 220 × 114.68 mm; voxel size: 2.75 mm^3^]. Number of volumes per participant varied due to self-paced questions (*M* = 241.6, s.d. = 9.93, range: 224–264). After obtaining functional scans, field maps were collected [TR: 200 ms, TE: 3.2 ms; maximum: 58 slices (optimum: 29 slices); voxel size: 2.75 mm^3^] for distortion correction. We acquired a structural 3D T1-FFE scan prior to the functional scans (TR: 7.9 ms, TE: 3.5 ms, flip angle: 8°; 155 transverse slices; FOV: 250 × 195.83 × 170.5 mm; voxel size: 1.10 mm^3^; duration: 4:11 min).

### Data pre-processing and analysis

#### Behavioural data analysis.

Behavioural data were analysed using R-3.5.1 (R Core Team, 2013). We used lme4 for multilevel analyses (Bates *et al.*, 2015) and ggplot2 for figures (Wickham, 2016). We analysed how mood varied as a function of predetermined feedback valence and self-rated applicability from trial-to-trial using a linear mixed model (Hox *et al.*, 2017), with intermediate feedback as reference category to which effects of positive and negative feedback were compared. Feedback valence categories and applicability ratings were specified on the first level; parental mood after each feedback word was included as outcome. All variables were mean-centred. All examined models include random effects for feedback valence as well as applicability. χ2-tests were used to test for significance of main and interaction effects.

To examine whether the impact of feedback on parental mood is dependent on parents’ *general* view of their child, feedback valence categories were included on the first level and general view scores on the second level with parental mood as outcome.

#### MRI data pre-processing.

MRI data were pre-processed and analysed using SPM12 (Wellcome Trust Centre for Neuroimaging, London, UK), implemented in MATLAB R2018b (MathWorks, Natick, MA). Both raw and pre-processed data were checked for quality, registration and movement. No participants moved more than one voxel (2.75 mm; *M* = 0.08 mm, s.d. = 0.04, range: 0.001–0.87). All functional scans were corrected for slice timing, corrected using field maps, unwarped and realigned, co-registered with the anatomical scan, normalized to MNI space using the DARTEL toolbox (Ashburner, 2007), resliced to 1.5 mm^3^ voxels and spatially smoothed with an 8 mm FWHM (full width half maximum) isotropic Gaussian kernel.

#### fMRI data analysis.

To examine neural responses to vicarious feedback and how they vary as a function of applicability, we defined a general linear model (GLM) that included separate regressors for onsets and durations of each predetermined feedback valence category. Feedback onset regressors were parametrically modulated by applicability ratings. The onsets and durations of the mood questions were set as regressors of no interest. The GLM further included six motion regressors to correct for head motion. For each subject, *t*-contrasts were computed to compare positive and negative feedback to each other and to intermediate feedback. Furthermore, *t*-contrasts were computed to test the main effect of applicability and the interaction between feedback valence and applicability. Subject-specific contrast images were submitted to group-level random-effects analyses. First, we examined BOLD responses to positive *vs* negative feedback (and the reverse contrast as well as *vs* intermediate feedback) and interactions with applicability using whole-brain *t*-test analyses. Whole-brain results were corrected for multiple comparisons using family-wise error (FWE) cluster correction at *P *< 0.05 (cluster-forming threshold of *P* < 0.001).

Next, we explored inter-individual differences in parents’ general view of their child using whole-brain regression analyses on the previously described contrasts with general view scores as a between-subjects regressor. In these analyses, applicability was not taken into account.

## Results

### Parental affective and neural responses to vicarious feedback

Parents’ mood increased after receiving positive (*b *= 0.66, SE* *= 0.05, *t *= 12.54) compared to intermediate feedback (*b* = 0.30, SE* *= 0.03, *t *= 0.93) and decreased after receiving negative (*vs* intermediate) feedback (*b *= −0.59, SE* *= 0.05, *t *= −11.56) about the child [χ2(2) = 214.1, *P* < 0.001].

**Fig. 2.**
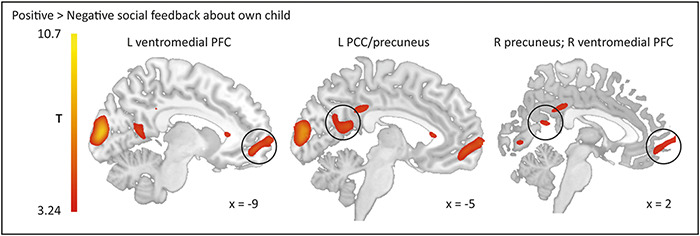
Activation in parental brains revealed by whole-brain regression analysis in response to positive as compared to negative feedback about own adolescent child [thresholded at *P* < 0.05 using family-wise error (FWE) cluster correction with a cluster-forming threshold of *P *< 0.001]. Abbreviations: PCC = posterior cingulate cortex; PFC = prefrontal cortex; L = left; R = right.

On a neural level, positive compared to negative vicarious feedback increased activity in vmPFC and posterior cingulate cortex (PCC)/precuneus (see Figure 2 and Table 2 for complete list of significant clusters). Compared to intermediate feedback, receiving positive vicarious feedback did not elicit any of the hypothesized regions (see Supplementary Table S3 for complete list of significant clusters).

**Table 2.** Brain regions revealed by whole-brain regression analysis in response to positive and negative vicarious feedback about own adolescent child

Contrast	MNI coordinates			Voxel test value	Cluster *P*-value	Cluster Size
Brain regions	*x*	*y*	*z*	*Z*		
*Positive > Negative*						
R Lingual gyrus	15	−75	−9	Inf	<0.001	7205
R Calcarine fissure	17	−75	9	6.05		
	20	−89	0	5.82		
L Calcarine fissure	−9	−93	11	6.92	<0.001	1781
L Middle occipital gyrus	−18	−105	9	3.30		
L Superior frontal gyrus, medial orbital (vmPFC)	−9	56	−6	4.44	<0.001	1237
L Superior frontal gyrus, medial	−6	69	3	4.29		
L Posterior cingulate gyrus	−5	−53	17	4.40	0.003	691
L Precuneus	−12	−56	11	3.97		
	−5	−65	24	3.45		
*Negative > Positive*						
R Superior frontal gyrus, medial (dmPFC)	8	48	32	6.52	<0.001	6198
	6	29	47	6.13		
	5	38	42	4.50		
R Superior frontal gyrus, dorsolateral	24	5	50	5.88	<0.001	2023
	27	5	65	4.22		
R Middle frontal gyrus	39	9	63	3.11		
R Inferior frontal gyrus, triangular part	62	24	20	5.86	<0.001	5287
	60	26	11	5.20		
R Inferior frontal gyrus, orbital part	48	30	−6	5.08		
L Inferior frontal gyrus, orbital part	−33	24	−8	5.54	<0.001	5488
	−48	30	−6	5.36		
L Inferior frontal gyrus, triangular part	−57	20	14	5.19		
R Superior parietal gyrus	15	−74	57	5.29	<0.001	1595
L Lingual gyrus	−9	−81	−9	5.25	<0.001	930
R Pallidum	17	5	−5	4.68	<0.001	917
R Caudate nucleus (DS)	15	6	12	4.31		
	15	14	8	4.17		
R Precentral gyrus	59	5	44	3.95	<0.001	979
R Middle frontal gyrus	48	15	45	3.83		
R Postcentral gyrus	65	−8	45	3.53		

*Notes*: Neural results are corrected for multiple comparisons using family-wise error (FWE) cluster correction at *P* < 0.05 with a cluster-forming threshold of *P *< 0.001. dmPFC, dorsomedial prefrontal cortex; DS, dorsal striatum; L, left; MNI, Montreal Neurological Institute; R, right; vmPFC, ventromedial prefrontal cortex; *Z, Z*-score.

**Fig. 3.**
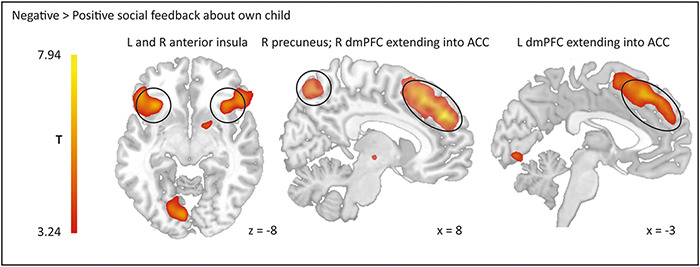
Activation in parental brain regions revealed by whole-brain regression analysis in response to negative as compared to positive feedback about own adolescent child. Neural results are corrected for multiple comparisons using family-wise error (FWE) cluster correction at *P* < 0.05 with a cluster-forming threshold of *P *< 0.001. Abbreviations: ACC = anterior cingulate cortex; dmPFC = dorsomedial prefrontal cortex; L = left; R = right.

Negative compared to positive vicarious feedback increased activity in a dmPFC cluster extending into ACC, an inferior frontal gyrus (IFG) cluster extending into AI and orbitofrontal gyrus (OFG), right superior parietal gyrus cluster extending into precuneus, and right dorsal striatum (DS; see Figure 3 and Table 2 for complete list of significant clusters). Compared to intermediate feedback, receiving vicarious negative feedback revealed no significant activations.

### Confound analyses

We ran additional analyses controlling for left-handedness, belief in the cover story, parental psychopathology and psychotropic medication use (see Supplementary Material 5). All findings remained significant except for the PCC/precuneus cluster, which failed to reach significance in the positive *vs* negative feedback contrast when adding left-handedness. Adding parental gender as covariate revealed differences in neural processing between mothers and fathers, see Supplementary Material 5.

### Associations with (in)applicability

**Fig. 4.**
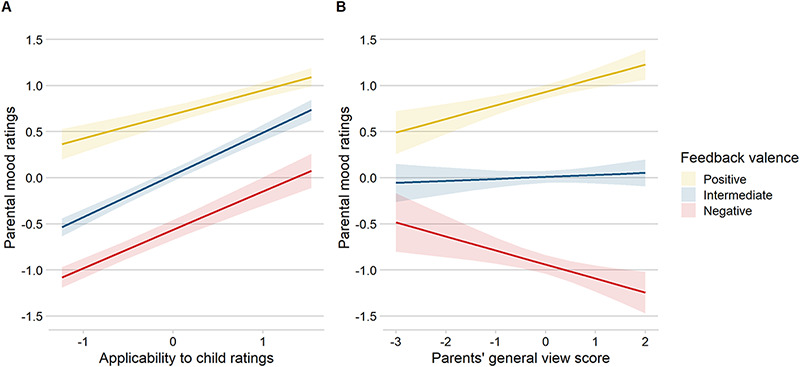
(A) Interaction effect of receiving positive (yellow), intermediate (blue) and negative (red) vicarious feedback about own child, which is not (−1) or very (1) applicable (mean-centred) on parental mood (mean-centred), *P* < 0.001. (B) Interaction effect of parents’ ‘general’ view of their child (mean-centred) on parental mood after receiving positive, intermediate and negative vicarious feedback about own child (mean-centred), *P* = 0.001.

More applicable feedback words increased parental mood, independent of valence [χ2(1) = 223.8, *P* < 0.001]. In addition, we found evidence for the expected interaction between feedback valence and applicability on parental mood [χ2(2) = 28.96, *P* < 0.001]. That is, when feedback words were regarded as inapplicable to their child, negative (*b *= 0.41, SE* *= 0.04, *t *= 10.92) and intermediate (*b *= 0.46, SE* *= 0.03, *t *= 15.13) feedback reduced mood the most, whereas mood was less affected by inapplicable positive feedback (*b *= 0.26, SE* *= 0.04, *t *= 6.82), see Figure 4A.

Whole-brain analyses testing for parametric effects of applicability and the feedback valence × applicability interaction did not result in any significant clusters that survived correction for multiple comparisons.

### Associations with parents’ general view

Inter-individual differences in parents’ general view of their child significantly impacted parental mood in response to vicarious social feedback [χ2(2) = 15.8, *P* = 0.001]. Viewing the child generally in a more positive light was associated with amplified mood responses, with more positive mood after positive feedback (*b *= 0.15, SE* *= 0.04, *t *= 3.93) and more negative mood after negative feedback (*b *= −0.15, SE* *= 0.05, *t *= −2.96). Parents’ general view of their child did not moderate mood after intermediate feedback on child (*b *= 0.02, SE* *= 0.03, *t *= 0.66), see Figure 4B.

**Fig. 5.**
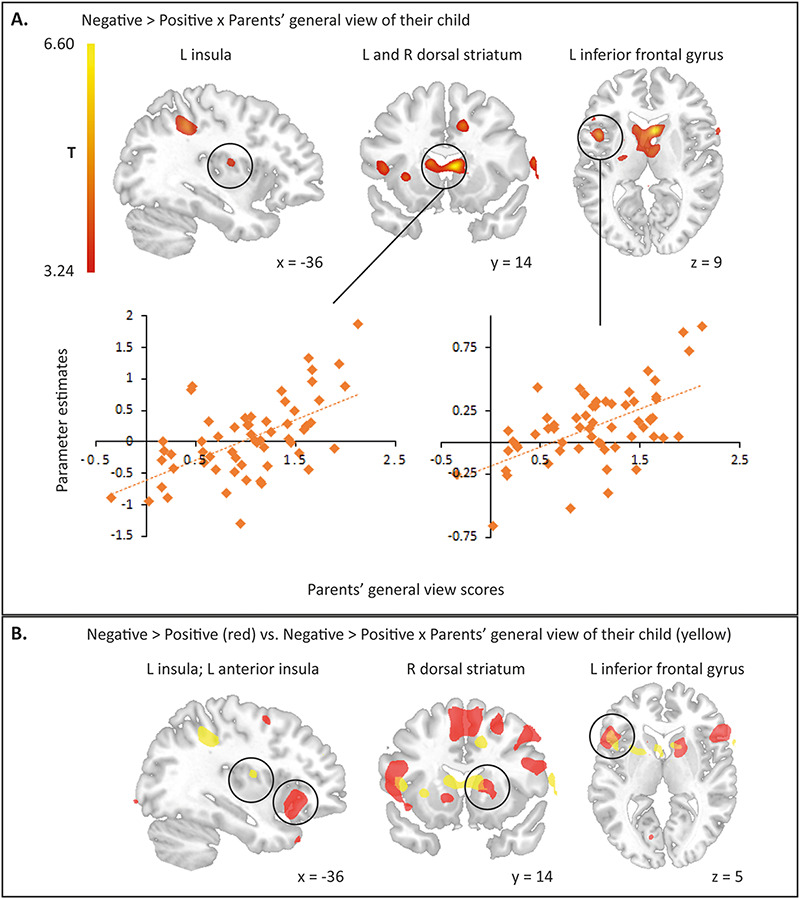
(A) The degree of parents’ general view of their child is positively related to increased activation in left insula, dorsal striatum (DS) and left inferior frontal gyrus (IFG), when parents receive negative compared to positive vicarious feedback about their child. Neural results are corrected for multiple comparisons using family-wise error (FWE) cluster correction at *P* < 0.05 with a cluster-forming threshold of *P *< 0.001. To visualize the interaction between inter-individual differences in parents’ general view and responses to negative (*vs* positive) vicarious feedback in significant brain clusters (i.e. DS cluster and IFG cluster, see also Table 3), we plotted average BOLD responses to negative (*vs* positive) feedback in brain clusters extracted against general view scores. Regression lines plotted for illustration purposes only. Abbreviations: L = left; R = right. (B) Overlap in brain areas of neural activation in R DS and L IFG (orange) in response to degree of parents’ general view of their child and areas associated with increased activation in negative *vs* positive feedback about their child. In red: neural activation related to receiving negative *vs* positive feedback about own child. In yellow: neural activation related to the degree of parents’ general view of their child.

A whole-brain analysis testing inter-individual differences in processing negative *vs* positive feedback using general view scores as between-subjects regressor revealed significant activation in a DS cluster extending into thalamus and left IFG cluster extending into insula (see Figure 5A and Table 3 for an overview of all clusters and Supplementary Table S5 for additional findings related to parents’ general view of their child). To examine the nature of this interaction, we plotted activity in both DS and IFG cluster as a function of parents’ general view separately for positive and negative feedback (*vs* implicit baseline). These plots suggest that this interaction seems to be driven by increased activity in these brain regions in response to negative feedback with increasingly positive general views parents have of their child (see Supplementary Figure S2). Interestingly, activity in right DS and left IFG overlapped with our findings related to receiving negative *vs* positive feedback (see Figure 5B). In none of the other contrasts significant activations were found.

**Table 3.** Brain regions, based on whole-brain regression analysis testing for inter-individual differences, that are associated with parents’ general view of their child in relation to negative *vs* positive feedback about their child

Contrast	MNI coordinates			Voxel test value	Cluster *P*-value	Cluster Size
Brain regions	*x*	*y*	*z*	*Z*		
*Negative > Positive × General view of child*						
R Caudate nucleus (DS)	6	14	9	5.49	<0.001	1678
L Caudate nucleus (DS)	−9	−5	17	4.32		
	−12	14	12	4.24		
L Inferior frontal gyrus, opercular part	−47	11	9	4.38	0.006	961
L Rolandic operculum	−50	−3	15	4.36		
L Inferior frontal gyrus, triangular part	−50	20	5	3.70		
L Inferior parietal gyrus	−39	−41	41	4.22	0.002	1161
L Postcentral gyrus	−53	−18	27	4.01		
L Inferior parietal gyrus	−53	−44	42	3.63		

*Notes*: Neural results are corrected for multiple comparisons using family-wise error (FWE) cluster correction at *P *< 0.05 with a cluster-forming threshold of *P *< 0.001. DS, dorsal striatum; L, left; MNI, Montreal Neurological Institute; R, right; *Z, Z*-score.

Additionally, when controlling for parental gender, left-handedness, parental belief in the cover story, parental psychopathology and psychotropic medication use, only minor coordinate changes in these neural findings were observed.

## Discussion

This study examined affective and neural responses in parents in response to praise and critique about their adolescent child. We investigated whether these responses were modulated by the (in)consistency of feedback with parents’ own perceptions of their child as well as by parents’ *general* view of their child. Our study yielded several novel and important findings. First, parental mood increased after receiving positive feedback and decreased after receiving negative feedback about their child. Parental mood decreased when vicarious feedback was inconsistent with their own perceptions of their child, especially after negative feedback (i.e. ‘misplaced criticism’). Additionally, *generally* viewing the child in a more positive light amplified mood responses to both positive and negative feedback. With respect to the neural responses, we found that parental vicarious social feedback processing engages brain regions involved in social salience processing (i.e. vmPFC, ACC and AI) and mentalizing (i.e. dmPFC, precuneus and IFG), which are similarly active when receiving social feedback about the self. More specifically, positive *vs* negative vicarious feedback increased activity in vmPFC and PCC/precuneus, whereas negative *vs* positive vicarious feedback elicited activity in ACC, AI, dmPFC, IFG and right precuneus. Finally, whereas the (in)applicability of each feedback word did not modulate the neural responses to that specific word, individual differences in parents’ *general* view revealed that receiving vicarious negative *vs* positive feedback increased activity in DS, thalamus, left IFG and left insula in parents who viewed their child more positively.

As expected, we found that parents are emotionally affected by both praise and criticism about their child. Our results demonstrate that feedback about one’s child activates brain regions related to social salience processing, similar to those found to be activated when receiving social feedback about the self. To be more specific, receiving praise about the child elicited activity in vmPFC, similar to prior studies investigating positive feedback (Izuma *et al.*, 2008; Davey *et al.*, 2010; Gunther Moor *et al.*, 2010; Korn *et al.*, 2012; Morelli *et al.*, 2015; Muscatell *et al.*, 2016; Will *et al.*, 2017, 2020; Kawamichi *et al.*, 2018; Schindler *et al.*, 2019). The vmPFC has been proposed to be central to social value computations (Morelli *et al.*, 2015; Muscatell *et al.*, 2016) and self-referential processing (Northoff *et al.*, 2006; Denny *et al.*, 2012). Research suggests that vmPFC may not support self-reflection *per se* but is also activated when inferring mental states of close others or more specifically, when making trait judgments about close others (Jenkins *et al.*, 2008; Van Overwalle, 2009; Heleven and Van Overwalle, 2019). The vmPFC might thus also respond to aspects beyond the self that have high personal value (D’Argembeau, 2013), in our case: one’s child, and may be important when parents process praise about their child. Alternatively, when parents received criticism about their child we found increased activity in ACC, AI, OFG and right DS, in accordance with previous studies looking at negative feedback about the self (Eisenberger *et al.*, 2011; Cacioppo *et al.*, 2013; Rotge *et al.*, 2015; Muscatell *et al.*, 2016; Will *et al.*, 2016; Kawamichi *et al.*, 2018; van Schie *et al.*, 2018; Schindler *et al.*, 2019; Fritz *et al.*, 2020). Our findings suggest that receiving *vicarious* negative feedback may elicit activation of a similar ‘neural alarm system’ as is activated by direct, personal negative feedback, in which ACC and AI are primarily involved (Eisenberger, 2012). These findings are also in line with prior studies showing that parents engage ACC and AI not only when experiencing negative affect themselves but also when empathizing with their child’s experiences of negative emotions or pain (Fan *et al.*, 2011; Feldman, 2015; Abraham *et al.*, 2018). In sum, parents clearly empathize with their child’s feelings when being socially evaluated and concurrently may also feel personally judged (Thai *et al.*, 2019). However, given that we did not include a self-condition in our paradigm, it remains unanswered to what extent parents *genuinely* vicariously experienced feedback about their child, i.e. actually felt and processed the feedback as if it concerned *themselves*, which needs further investigation.

Additionally, vicarious praise about own child elicited PCC/precuneus activity, whereas vicarious criticism activated dmPFC, right precuneus and IFG, which is also in line with previous social feedback studies (Muscatell *et al.*, 2016; Kawamichi *et al.*, 2018; van Schie *et al.*, 2018). These areas are commonly found to be related to mentalizing processes (Van Overwalle, 2009; Schurz *et al.*, 2014; Molenberghs *et al.*, 2016). Correspondingly, activation in these areas has also been found when parents make judgments about traits of their offspring (Laurita *et al.*, 2019) or when mothers receive rewards for their offspring (Spaans *et al.*, 2018). In our study it is not distinguishable, however, whether the activation of the mentalizing network reflects parental reflections and considerations on the feedback providers (in our case, research assistants), on their child, or both. Either way, it seems plausible that mentalizing processes are crucial when parents’ own child is being evaluated, in order to process and act aptly upon the provided feedback. It should be noted, however, that the PCC/precuneus cluster in response to vicarious praise failed to reach significance when adding left-handedness, and hence replications of these findings are warranted.

Another key finding is that parents’ perceptions of their child’s qualities and flaws greatly affected their responses to vicarious feedback. Parental mood decreased when feedback words were inconsistent with existing parental perceptions, regardless of feedback valence. Especially for subjectively ‘misplaced criticism’ about their child, parental mood reduced significantly, which is remarkably similar to previous research investigating the impact of applicability of social feedback about the self (van Schie *et al.*, 2018). Interestingly, we did not find any brain region where activity was moderated by (in)applicability of feedback words, in contrast with van Schie *et al.* (2018), who found heightened left precuneus activation in response to more applicable feedback. Given that in the study by van Schie *et al.* (2018) applicability of feedback was assessed directly *after* the task, these applicability ratings may have been influenced by the provided feedback itself, which may explain the different outcomes. Furthermore, processing *vicarious* feedback about one’s child may generally yield more complex cognitive processes (i.e. thinking about the child, the feedback provider(s) *and* one’s own perceptions) as compared to feedback about oneself. Hence, subtle differences such as activation related to *(in)applicability* of feedback may therefore be more difficult to capture in a vicarious paradigm.

*Generally* viewing their child through more ‘rosy’ glasses was associated with both amplified mood responses to praise as well as critique about the child and neural responses to critique. The more parents view their child in a positive light, the more vicarious critique elicited activity in left IFG, left insula, DS and thalamus. Interestingly, IFG and DS activation overlapped with our clusters activated in response to vicarious negative feedback, indicating that these responses seem to be augmented in parents with more positive views on their child. Given that the dorsal part of striatum is related to updating action values (Balleine *et al.*, 2007; Kim *et al.*, 2009; Ruff and Fehr, 2014) and thalamus plays an important role in integrating information and regulating cognitive efforts (Schiff *et al.*, 2013; Bell and Shine, 2016; Jiang *et al.*, 2018), this may suggest that parents who view their child more positively may engage in more effortful information and mentalizing processing when their child is being criticized and might try to uphold their positive view of their child (Vogels and Perunovic, 2020). As receiving critique about their child violates expectations to a larger extent in parents with more ‘rosy glasses’, critique might be emotionally more salient, which may also be reflected in heightened insula activation (Menon and Uddin, 2010). Thus, especially parents who view their child more positively seem to be most affected by social feedback about their child, both on a behavioural and a neural level.

A remaining question is whether ‘rosy’ glasses are always advantageous or whether they can also have *disadvantageous* side effects for parents and their children. This certainly warrants further investigation (van Schie *et al.*, 2020). The evoked emotions in parents when receiving feedback about their child can result in a large variety of outcomes. Parents might—depending on the strength of their emotions and regulation skills—minimize (the importance of) the given critique, blame the criticizer, criticize the child *themselves* or (over)praise the child (Brummelman *et al.*, 2017; Brummelman and Thomaes, 2017; Vogels and Perunovic, 2020). If parents who show heightened susceptibility to vicarious feedback also *express* negative emotions more strongly when confronted with critique about their child, this may also shape the *child’s* feelings about this particular critique and corresponding self-views, and in the long run, the child’s global evaluation of the self, i.e. self-esteem (Harter, 2015; Brummelman and Thomaes, 2017). An interesting direction for future work would be to focus on the underlying neural mechanisms of individual differences in parental *behavioural* reactions to vicarious feedback. Longitudinal designs might also give insights into the role parents potentially play in the development of self-views and self-esteem of their children during adolescence, given that negative self-views and low self-esteem are commonly found as predictors of mental health problems, such as depression (Swann *et al.*, 2007).

To conclude, our study has several strengths and also some limitations. First, we employed a unique and ecologically valid paradigm, using realistic social feedback, a credible and comprehensive cover story that most parents believed and a sensitive debriefing method. Second, incorporating parents’ own perceptions of their child in the design (assessed *prior* to the actual task) substantially adds to the literature on social feedback and corresponding self-views. Third, we recruited a substantial sample of parents of non-clinical adolescents, including both mothers *and* fathers. fMRI research on parents of adolescents, and fathers in particular, is still scarce, and our design allows for more generalizable conclusions. Yet, whereas there are indications for differential activation patterns in mothers and fathers, larger sample sizes are needed to draw valid conclusions on differences in neural responses to vicarious feedback between mothers and fathers. Another limitation is that we were not able to elucidate the mental processes of parents when experiencing vicarious feedback about their child, i.e. whether they experienced the feedback as if it concerns themselves, and how empathy for their child, and considerations about the feedback and feedback providers feed in. Finally, our measure of parents’ general view was a new construct, which has to be further validated and replicated.

## Conclusion

Taken together, the present study provides—to our knowledge—the first investigation of how parents experience vicarious praise and critique about their child in terms of affective and neural responses. Our results provide evidence that parents—depending on their own perceptions of their child—are emotionally affected by social feedback about their child and engage similar brain regions as those involved in processing feedback directed at the self. Although the parents generally appraised their child positively, parents who view their child with more ‘rosy’ glasses may be especially sensitive to vicarious praise and critique. Insights in the way parents view and react to compliments and critique about their child may be highly relevant for parenting practices and interventions, as targeting awareness of parents’ own perceptions and reactions to feedback might potentially be an important pillar in parenting interventions for adolescent mental health problems, such as clinically low self-esteem and depression.

## Supplementary Material

nsab004_Supp
