# Supplementary material for: Vicarious praise and pain: parental neural responses to social feedback about their adolescent child
Source: Soc Cogn Affect Neurosci. 2021 Jan 12;16(4):406–17. doi: 10.1093/scan/nsab004 (PMC7990067; doi:10.1093/scan/nsab004)
Supplement: nsab004_Supp [file nsab004_supp.zip › supplementary.docx]

**Supplementary Material**

1. ***Participants’ psychopathology and medication use***

Parents were screened on current axis I disorders using the Mini-International Neuropsychiatric Interview (MINI), Dutch version 5.0.0 (Sheehan et al., 1998; Van Vliet et al., 2000). In total, six parents (i.e., 10%) fulfilled criteria of a current axis I disorder (mood disorder: *n* = 1; anxiety disorder: *n* = 2; obsessive-compulsive disorder: *n* = 1; substance abuse: *n* = 1; mania and comorbid generalized anxiety disorder, and alcohol and drugs abuse: *n* = 1). Moreover, five parents (i.e., 8.3%) reported psychotropic medication use at the day of scanning (SSRIs: *n* = 3; SNRIs: *n* = 1; Methylphenidate: *n* = 1) and six parents (i.e., 10%) used medication for physical ailments (ACE-inhibitor: *n* = 1; Antioestrogen: *n* = 1; β-adrenergic receptor agonist: *n* = 1; levothyroxine: *n* = 1; NSAIDs: *n* = 1; Statins: *n* = 1).

1. ***Descriptive statistics and validation of vicarious feedback words***

All words used in the vicarious social feedback task are shown in Supplementary Table S1. Our word sample is based on van Schie and colleagues (2018). However, we replaced words that were less understandable and/or common for adolescents, as indicated in a pilot study (*n* = 10) with adolescents. Word length was equal across feedback valence categories [*F*(2,42) = 1.57, *p* = .219, ns], as well as word frequency of occurrence in the Dutch language (based on Keuleers et al., 2010) per feedback valence category [*F*(2,42) = 1.26, *p* = .294, ns]. Additionally, we used 4 positive feedback fillers: *‘Attent’* (Attentive), *‘Plezierig’* (Pleasurable), *‘Slim’* (Smart) and *‘Sociaal’* (Social) at the beginning and end of the task. These words are not taken into account in any analyses.

Parents rated positive feedback words (*b* = 0.96, *SE* = 0.04, *t* = 26.87) as more positive than intermediate feedback words (*b* = 0.05, *SE* = 0.02, *t* = 2.82), which were rated as more positive than negative feedback words (*b* = -1.11, *SE* = 0.04, *t* = -30.27) [χ2(2) = 1397.4, *p* < .001], validating the predetermined valence categories.

Moreover, parents rated positive feedback words (*b* = 0.96, *SE* = 0.04, *t* = 21.61) as more applicable to their child than intermediate words, (*b* = 0.04, *SE* = 0.02, *t* = -1.83) and negative feedback words (*b* = -0.84, *SE* = 0.04, *t* = -20.07). Negative words were rated as less applicable to their child than intermediate words [χ2(2) = 764.88, *p* < .001], illustrating parents’ overall rosy glasses.

**Supplementary Table S1.** Positive, intermediate and negative vicarious feedback words used in the vicarious social feedback task in Dutch (left) and English (right)

| *Positive* | | *Intermediate* | | *Negative* | |
| --- | --- | --- | --- | --- | --- |
| Aardig | Kind | Chaotisch | Chaotic | Arrogant | Arrogant |
| Blij | Happy | Eigenwijs | Stubborn | Bazig | Bossy |
| Creatief | Creative | Emotioneel | Emotionally | Egoïstisch | Selfish |
| Eerlijk | Honest | Hard | Harsh | Gemeen | Mean |
| Energiek | Energetic | Impulsief | Impulsive | Irritant | Annoying |
| Gezellig | Sociable | Kritisch | Critical | Laf | Cowardly |
| Grappig | Funny | Nerveus | Nervous | Lui | Lazy |
| Intelligent | Intelligent | Netjes | Neat | Naar | Nasty |
| Lief | Sweet | Onhandig | Clumsy | Onbetrouwbaar | Unreliable |
| Nieuwsgierig | Curious | Onzeker | Insecure | Saai | Boring |
| Respectvol | Respectful | Praatgraag | Talkative | Sloom | Slow |
| Spontaan | Spontaneously | Raar | Weird | Somber | Gloomy |
| Vriendelijk | Friendly | Rustig | Quiet | Stom | Stupid |
| Vrolijk | Cheerful | Serieus | Serious | Vals | Vicious |
| Wijs | Wise | Streng | Strict | Vervelend | Unpleasant |

1. ***Manipulation check interview***

Once participants were outside the scanner, a manipulation check interview was held to assess whether they believed the cover story that feedback was provided by research assistants. The questions were asked verbally and answers were audio recorded, see Supplementary Table S2 for the specific questions.

Questions 1-5 revealed whether parents had any doubts about the task setup without explicitly indicating that there was need for doubt. Questions 6-8 assessed the degree of doubt (if any doubt was expressed by the participant). We categorised participants in two groups: believers (I) were convinced by the task setup and answered questions 6-8 with serious thought or minor doubt, showing expressions of belief; non-believers (II) showed spontaneous expressions of disbelief during questions 1-5. Three researchers independently judged whether each participant should be assigned to the believer or non-believer category, by listening to the recorded voice memos. Fleiss’ kappa showed that the interrater agreement was good [κ = .70, *p* < .001]. The majority of parents (*n* = 50, 83.3%) believed the cover story (see *Methods* section in main text).

**Supplementary Table S2.** Verbally asked questions during manipulation check interview of vicarious social feedback task in Dutch (left) and English (right)

| *Question* | |  | |  |
| --- | --- | --- | --- | --- |
| 1 | In hoeverre bent u het eens met de feedbackwoorden die u over uw kind gehoord heeft van de onderzoeksassistenten uit ons onderzoeksteam? | | To what extent do you agree with the feedback words you have received about your child from the research assistants? | |
| 2 | In hoeverre denkt u dat de indruk die de onderzoeksassistenten uit ons onderzoeksteam van uw kind hebben klopt? | | To what extent do you think the impression that the research assistants have of your child is correct? | |
| 3 | In hoeverre denkt u dat de onderzoeksdag voldeed om een volledige indruk van uw kind te krijgen? | | To what extent do you think the research day sufficed to get a complete impression of your child? | |
| 4 | Wat is uw indruk van de onderzoeksassistenten die uw kind de feedback hebben gegeven? | | What is your impression of the research assistants who gave your child feedback? | |
| 5 | Heeft de feedback u emotioneel geraakt? Waarom wel of niet? | | Were you emotionally affected by the feedback? Why (not)? | |
| 6 | Hoe zeker bent u ervan dat de onderzoeksassistenten uw kind feedback heeft gegeven? | | How confident are you that the research assistants gave your child feedback? | |
| 7 | Dacht u dat de hele tijd? Vanaf wanneer wel of niet? | | Did you have this feeling during the whole task? Or at what point changed this feeling? | |
| 8 | Wat waren redenen om te twijfelen aan de opzet? | | What were reasons to doubt the task setup? | |

1. ***Neural findings in response to positive vs. intermediate vicarious feedback***

**Supplementary Table S3.** Brain regions revealed by whole-brain regression analysis in response to positive vs. intermediate vicarious feedback about own child

| *Contrast* | | MNI coordinates | | | Voxel test value | Cluster | Cluster |
| --- | --- | --- | --- | --- | --- | --- | --- |
| Brain regions |  | **x** | **y** | **z** | **Z** | ***p*-value** | **size** |
| *Positive > Intermediate* | | | | | | | |
| R Lingual gyrus | | 15 | -71 | -6 | 6.40 | <.001 | 3760 |
| R Superior occipital gyrus | | 24 | -81 | 33 | 5.08 |  |  |
| R Calcarine fissure | | 26 | -74 | 14 | 4.50 |  |  |
| L Precentral gyrus | | -39 | -26 | 69 | 5.18 | <.001 | 1226 |
|  | | -42 | -21 | 57 | 4.61 |  |  |
| L Cuneus | | -11 | 92 | 15 | 5.00 | .005 | 640 |

Notes: Neural results are corrected for multiple comparisons using Family-wise Error (FWE) cluster-correction at *p* < .05 with a cluster-forming threshold of *p* < .001. Abbreviations: L = left; R = right; MNI = Montreal Neurological Institute; Z = Z-score.

1. ***Confounds and exploration of gender differences***

Adding gender, current parental psychopathology or belief in the cover story as covariate did not change any behavioural results. In general, when taking current psychopathology, belief in the cover story, psychotropic medication use or left-handedness separately into account in the neural analyses, minor changes in peak coordinates of neural results were observed. Specifically, when adding left-handedness, the PCC/precuneus cluster failed to reach significance in the positive vs. negative vicarious feedback contrast. Moreover, if belief in the cover story was added as covariate, the last three clusters in the negative vs. positive vicarious feedback contrast, i.e. lingual gyrus, pallidum/DS, and precentral gyrus/middle frontal gyrus clusters, failed to reach significance (see Table 2 for relevant clusters).

Adding gender as covariate revealed some major differences between fathers (*n* = 25) and mothers (*n* = 35) in neural results. Overall, fathers showed more cluster-activation to positive vs. negative vicarious feedback, whereas mothers showed more cluster-activation to negative as compared to positive vicarious feedback, see Supplementary Table S4.

Specifically, when receiving positive vs. negative vicarious feedback about their child, activation in vmPFC and PCC/precuneus clusters failed to reach significance in mothers, whereas in fathers these clusters remained significant, plus additional activation in right PCC was found. See Supplementary Table S4 for complete overview of all significant clusters.

On the other hand, when receiving negative vs. positive vicarious feedback, activation in AI, ACC, OFG, right DS, dmPFC, right precuneus and IFG all remained significant in mothers, whereas in fathers only a dmPFC cluster extending into ACC, and right IFG cluster remained significant. See Supplementary Table S4 for complete overview of significant clusters.

**Supplementary Table S4.** Brain regions revealed by whole-brain regression analysis in response to positive and negative vicarious feedback about own child with gender added as covariate (0 = fathers, 1 = mothers)

| *Contrast* | MNI coordinates | | | Voxel test value | Cluster | Cluster |
| --- | --- | --- | --- | --- | --- | --- |
| Brain regions | **x** | **y** | **z** | **Z** | ***p*-value** | **size** |
| *Positive > Negative* |  |  |  |  |  |  |
| R Lingual gyrus | 14 | -77 | -11 | 6.80 | <.001 | 16872 |
|  | 8 | -86 | -11 | 6.46 |  |  |
| R Calcarine fissure | 20 | -93 | 5 | 6.12 |  |  |
| L Calcarine fissure | -9 | -93 | 12 | 6.07 | <.001 | 2331 |
| L Middle occipital gyrus | -15 | -105 | 2 | 4.28 |  |  |
|  | -23 | -102 | 17 | 3.20 |  |  |
| L Superior frontal gyrus, medial orbital (vmPFC) | -9 | 57 | -5 | 4.47 | .001 | 941 |
| L Superior frontal gyrus, medial | -14 | 62 | 2 | 4.12 |  |  |
|  | -3 | 72 | 2 | 3.68 |  |  |
| L Postcentral gyrus | -45 | -27 | 60 | 4.29 | <.001 | 1104 |
| L Precentral gyrus | -41 | -21 | 66 | 4.07 |  |  |
|  | -33 | -27 | 71 | 3.60 |  |  |
| L Superior parietal gyrus | -17 | -50 | 75 | 4.04 | <.001 | 1307 |
| L Precuneus | -3 | -44 | 65 | 3.89 |  |  |
|  | -8 | -44 | 75 | 3.81 |  |  |
| *Positive > Intermediate* |  |  |  |  |  |  |
| R Superior occipital gyrus | 23 | -90 | 38 | 5.42 | <.001 | 5804 |
| R Middle temporal gyrus | 36 | -56 | 14 | 4.88 |  |  |
| L Superior occipital gyrus | 26 | -83 | 33 | 4.83 |  |  |
| L Postcentral gyrus | -47 | -27 | 65 | 4.78 | .002 | 1400 |
| L Precentral gyrus | -39 | -26 | 69 | 4.64 |  |  |
|  | -42 | -21 | 57 | 4.19 |  |  |
| *Negative > Positive* |  |  |  |  |  |  |
| R Inferior frontal gyrus, triangular part | 63 | 24 | 12 | 5.03 | .002 | 1124 |
|  | 53 | 26 | 12 | 4.18 |  |  |
| R Inferior frontal gyrus, opercular part | 63 | 20 | 32 | 3.20 |  |  |
| R Superior frontal gyrus, medial (dmPFC) | 6 | 47 | 32 | 4.84 | .001 | 1216 |
|  | 6 | 32 | 45 | 4.44 |  |  |
| L Superior frontal gyrus, medial | -3 | 38 | 44 | 4.01 |  |  |

Notes: Neural results are corrected for multiple comparisons using Family-wise Error (FWE) cluster-correction at *P* < 0.05 with a cluster-forming threshold of *P* < 0.001. Abbreviations: dmPFC = dorsomedial prefrontal cortex; vmPFC = ventromedial prefrontal cortex; L = left; R = right; MNI = Montreal Neurological Institute; Z = Z-score.

We furthermore explored whether fathers and mothers differed in their affective responses. Predetermined valence categories (i.e. positive, intermediate and negative feedback) and self-reported applicability ratings for each feedback word were specified on the first level and gender was included on the second level. Parental mood after each feedback word was included as outcome.

No main effect of gender was found (*b* = 0.12, *SE* = 0.06, *t* = 1.85) [χ2(1) = 0.11, *p* = .74, ns]. We did find an interaction effect of valence category*gender [χ2(2) = 8.53, *p* = .01], indicating that mothers had a more positive mood after receiving positive vicarious feedback about their child (*b* = 0.76, *SE* = 0.07, *t* = 11.53), as compared to fathers (*b* = -0.26, *SE* = 0.10, *t* = -2.50), whereas they did not differ in response to negative and intermediate and feedback. Moreover, no three-way interaction effect of valence category*applicability*gender on parental mood was found [χ2(2) = 5.01, *p* = .08, ns], see Supplementary Figure S1.

Given we had no a priori hypotheses about gender differences combined with the small group size, these differences should be interpreted with care and replicated in larger samples.

**Supplementary Figure S1.** Interaction effect of receiving positive, intermediate and negative vicarious feedback about own child which is not (-1) or very (1) applicable (mean-centred) on parental mood (mean-centred), separately for mothers (left panel) and fathers (right panel). The observed effect is not significantly different for mothers as compared to fathers, *p* = .08, ns. Moreover, mothers reported a more positive mood after receiving positive vicarious feedback about their child as compared to fathers, *p* = .01.

***
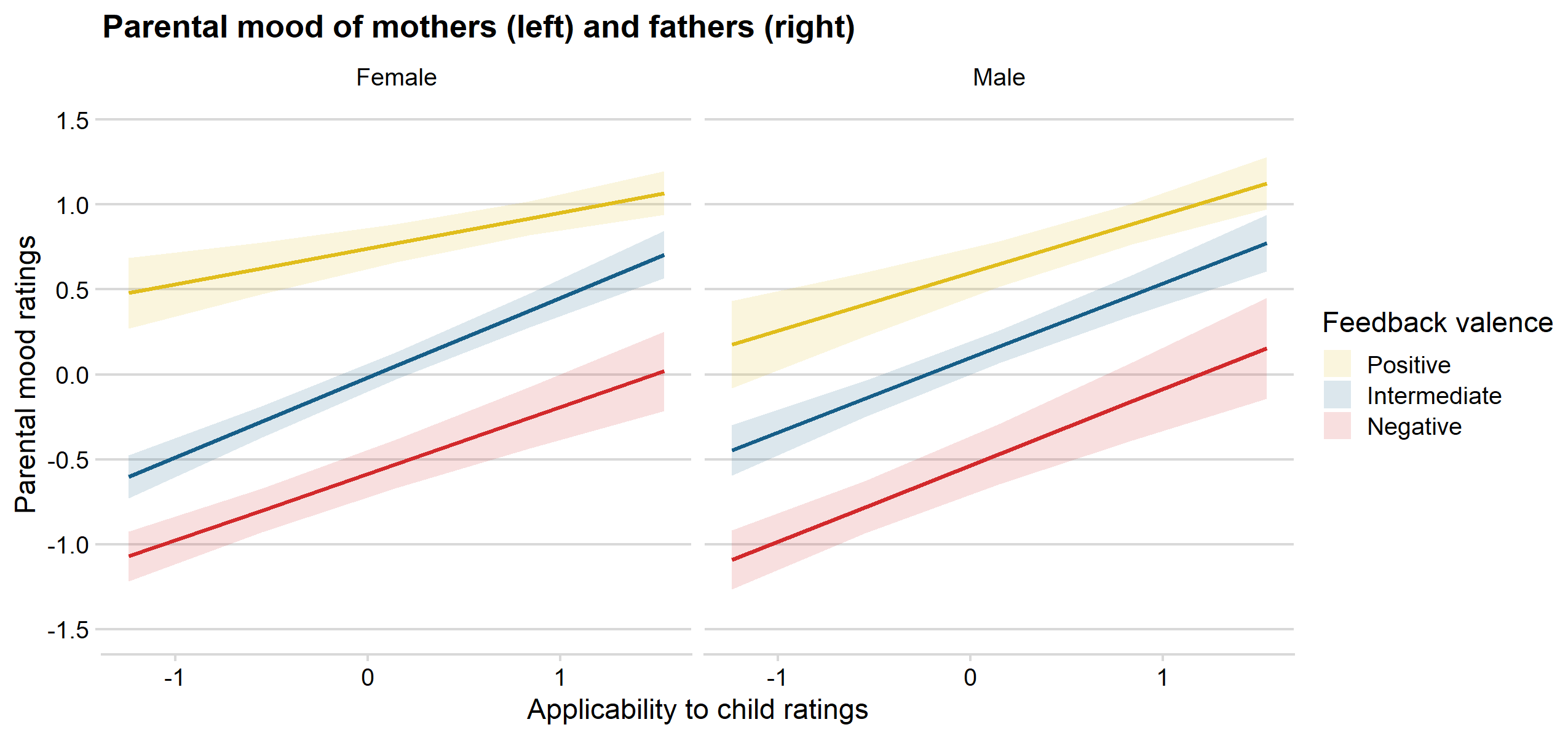
***

1. ***Additional neural findings related to the level of parents’ general view of their child***

**Supplementary Table S5.** Brain regions, based on whole-brain regression analysis testing for inter-individual differences, that are associated with parents’ general view of their child in relation to vicarious feedback about their child

| *Contrast* | MNI coordinates | | | Voxel test value | Cluster | Cluster |
| --- | --- | --- | --- | --- | --- | --- |
| Brain regions | **x** | **y** | **z** | **Z** | ***p*-value** | **size** |
| *Intermediate > Positive x General view of child* | |  |  |  |  |  |
| L Inferior parietal gyrus | -47 | -41 | 44 | 4.40 | .039 | 654 |
|  | -38 | -39 | 42 | 3.93 |  |  |
|  | -29 | -44 | 44 | 3.28 |  |  |
| *Negative x General view of child* |  |  |  |  |  |  |
| L Middle frontal gyrus | -27 | 11 | 42 | 4.75 | .019 | 697 |
| L Superior frontal gyrus, dorsolateral | -23 | 20 | 56 | 4.02 |  |  |
|  | -17 | 14 | 53 | 3.59 |  |  |
| *Intermediate x General view of child* |  |  |  |  |  |  |
| L Middle frontal gyrus | -29 | 12 | 42 | 4.92 | <.001 | 1846 |
| L Superior frontal gyrus, dorsolateral | -21 | 2 | 47 | 4.49 |  |  |
|  | -20 | 42 | 33 | 4.32 |  |  |

Notes: Neural results are corrected for multiple comparisons using Family-wise Error (FWE) cluster-correction at *p* < .05 with a cluster-forming threshold of *p* < .001. Abbreviations: L = left; R = right; MNI = Montreal Neurological Institute; Z = Z-score.

**Supplementary Figure S2.** The degree of parents’ general view of their child in relation to activity in DS (**A**) and IFG (**B**) cluster separately for positive and negative feedback (vs. implicit baseline). To visualise whether the interaction between inter-individual differences in parents’ general view and responses to negative vs. positive vicarious feedback in significant brain clusters (i.e. DS cluster (**A**) and IFG cluster (**B**), see also Table 3) were driven by a greater activity in response to negative feedback, or a reduced activity in response to positive feedback, we plotted average BOLD-responses in brain clusters extracted against general view scores separately for positive feedback (left graph in green) and negative feedback (right graph in red) vs. implicit baseline. Regression lines plotted for illustration purposes only. Abbreviations: L = left; R = right.

**
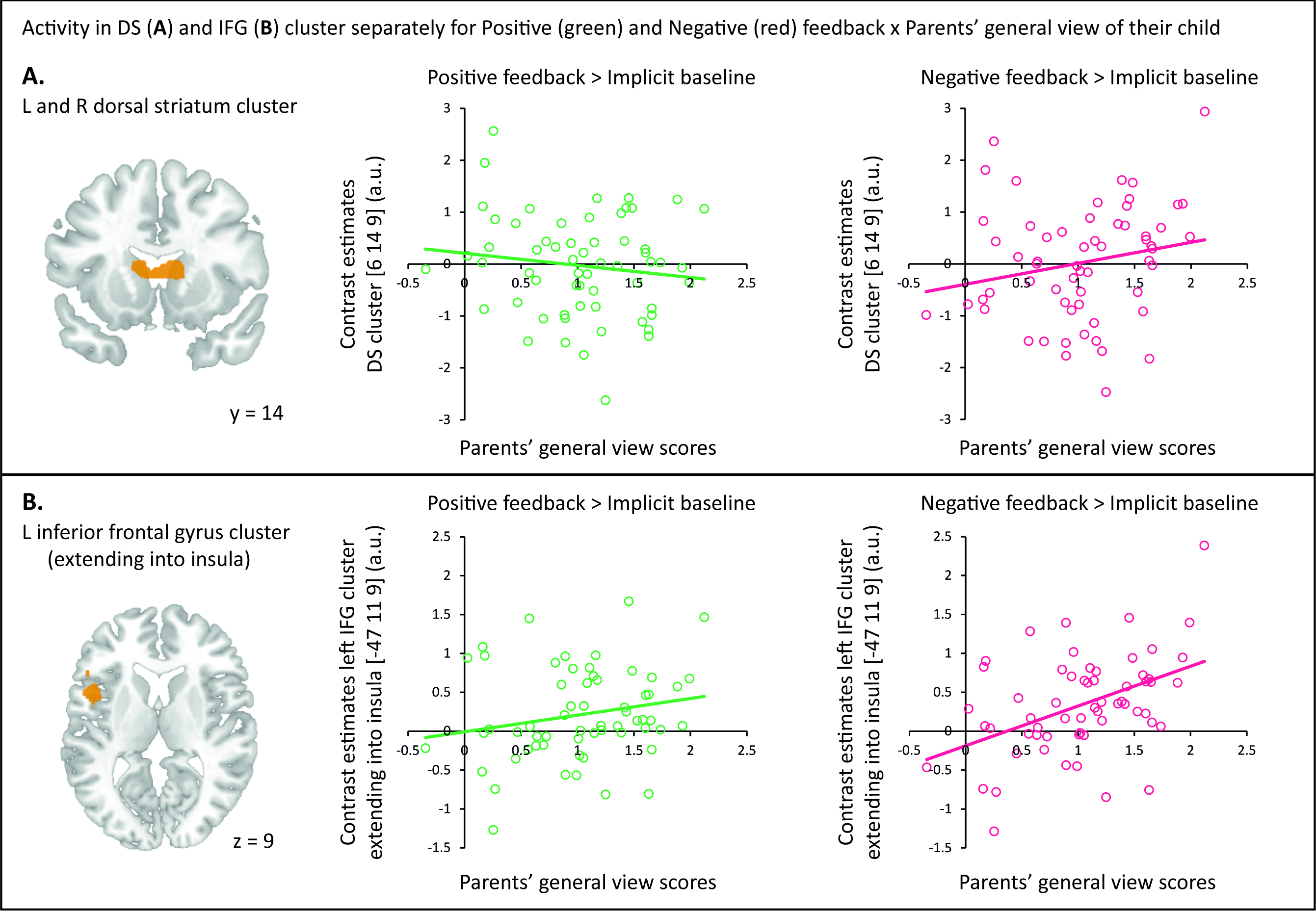
**

**Supplemental References**

Keuleers, E., Brysbaert, M., & New, B. (2010). SUBTLEX-NL: a new measure for Dutch word frequency based on film subtitles. *Behav Res Methods*, *42*(3), 643-650. <https://doi.org/10.3758/BRM.42.3.643>

Sheehan, D. V., Lecrubier, Y., Sheehan, K. H., Amorim, P., Janavs, J., Weiller, et al. (1998). The Mini-International Neuropsychiatric Interview (M.I.N.I.): the development and validation of a structured diagnostic psychiatric interview for DSM-IV and ICD-10. *J Clin Psychiatry*, *59 Suppl 20*, 22-33;quiz 34-57. <https://www.ncbi.nlm.nih.gov/pubmed/9881538>

van Schie, C. C., Chiu, C. D., Rombouts, S., Heiser, W. J., & Elzinga, B. M. (2018). When compliments do not hit but critiques do: an fMRI study into self-esteem and self-knowledge in processing social feedback. *Soc Cogn Affect Neurosci*, *13*(4), 404-417. <https://doi.org/10.1093/scan/nsy014>

Van Vliet, I., Leroy, H., & Van Megen, H. (2000). MINI Internationaal Neuropsychiatrisch Interview. *Nederlandse versie*, *5*(0).
